# Extracellular vesicles rejuvenate the microenvironmental modulating function of recipient tissue-specific mesenchymal stem cells in osteopenia treatment

**DOI:** 10.3389/fendo.2023.1151429

**Published:** 2023-03-22

**Authors:** Soichiro Sonoda, Takayoshi Yamaza

**Affiliations:** Department of Molecular Cell Biology and Oral Anatomy, Kyushu University Graduate School of Dental Science, Fukuoka, Japan

**Keywords:** mesenchymal stem cells, extracellular vesicles, microenvironmental modulation, telomerase, osteopenia

## Abstract

Systemic transplantation of mesenchymal stem cells (MSCs), such as bone marrow MSCs (BMMSCs) and stem cells from human exfoliated deciduous teeth (SHED), is considered a prominent treatment for osteopenia. However, the mechanism of action of the transplanted MSCs has been poorly elucidated. In the recipient target tissue, including bone and bone marrow, only a few donor MSCs can be detected, suggesting that the direct contribution of donor MSCs may not be expected for osteopenia treatment. Meanwhile, secretomes, especially contents within extracellular vesicles (EVs) released from donor MSCs (MSC-EVs), play key roles in the treatment of several diseases. In this context, administrated donor MSC-EVs may affect bone-forming function of recipient cells. In this review, we discuss how MSC-EVs contribute to bone recovery recipient tissue in osteopenia. We also summarize a novel mechanism of action of systemic administration of SHED-derived EVs (SHED-EVs) in osteopenia. We found that reduced telomerase activity in recipient BMMSCs caused the deficiency of microenvironmental modulating function, including bone and bone marrow-like niche formation and immunomodulation in estrogen-deficient osteopenia model mice. Systemic administration of SHED-EVs could exert therapeutic effects on bone reduction *via* recovering the telomerase activity, leading to the rejuvenation of the microenvironmental modulating function in recipient BMMSCs, as seen in systemic transplantation of SHED. RNase-preconditioned donor SHED-EVs diminished the therapeutic benefits of administrated SHED-EVs in the recipient osteopenia model mice. These facts suggest that MSC-EV therapy targets the recipient BMMSCs to rejuvenate the microenvironmental modulating function *via* telomerase activity, recovering bone density. We then introduce future challenges to develop the reproducible MSC-EV therapy in osteopenia.

## 1 Introduction

Owing to the osteoblast differentiation capacity, mesenchymal stem cells (MSCs) have been considered a promising source for degenerative skeletal disease, such as osteopenia. Recent studies show that systemic transplantation of MSCs rescues the bone mineral reduction in both *Fas*-mutated MRL/*lpr* and ovariectomy-induced estrogen-deficient (OVX) mice (1, 2). However, despite the persisting therapeutic effects of systemic MSC transplantation, infrequent engraftments of donor MSCs are recognized in the recipient target tissues, including bone and bone marrow, suggesting that the direct contribution of donor MSCs does not fully explain the solid mechanism of systemic MSC transplantation in osteopenia.

Increasing reports suggest that donor MSCs exert therapeutic effects *via* releasing secretomes, such as cytokines, chemokines, and growth factors (3). Notably, recent advances in the biology of extracellular vesicles (EVs) lead to an increased understanding of intercellular communication between donor cells and recipient cells in MSC therapy (4). EV-containing bioactive molecules, such as RNAs, DNA, lipid, and proteins, are transferred to affect the functions of recipient cells (5, 6). In addition, current advances in the procedure of EV isolation promote the number of studies on EV therapy as a novel promising option (4).

As of February 2022, according to ClinicalTraials.gov (https://clinicaltrials.gov) as a reference, there is no clinical trial using MSC-derived EVs (MSC-EVs) for treating osteopenia/osteoporosis, even though less than ten clinical studies of MSC therapy are conducted in osteopenia/osteoporosis.

Previous preclinical studies nominate the various mechanisms of MSC-EV therapy. On the other hand, the precise criteria to clarify the quality of MSC-EVs in osteopenia treatment remains unclear because the definitive molecules and their targets are not identified. Here, we review the current understanding of molecular actions to recover bone reduction by systemic administration of MSC-EVs in osteopenia. We further introduce a novel mechanism of MSC-EV therapy for osteopenia treatment; the administrated MSC-EVs are involved in rejuvenizing the tissue-specific microenvironmental modulating function of recipient bone marrow MSCs (BMMSCs) through promoting *Tert* gene expression and telomerase activity, resulting in exerting the bone mineral recovery in osteopenia model animals.

Based on current preclinical knowledge, as a future perspective, we propose that focusing on the microenvironmental modulating function of recipient BMMSCs as a target of MSC-EV therapy for osteopenia enables to establish a criterion for the quality control of MSC-EVs to achieve MSC-EV therapy a standard option for osteopenia.

## 2 Discovery and property of MSCs

In the 1970s, a Russian biologist Alexander J. Friedenstein first discovers a clonogenic cell population in bone marrow stromal cells, colony-forming units-fibroblast (CFU-F) (7). The later studies report that CFU-F-forming stromal cells exhibit spindle-shaped and plastic-adherent features and show the properties of *in vitro* and *in vivo* osteoblast differentiation (8). Sequential transplantation assay also reveals the self-renewal capacity of the bone marrow stromal cells (9, 10). Further investigations identify that the bone marrow stromal cells exhibit the multipotency into mesenchymal tissue-specific cells, such as adipocytes and chondrocytes, as well as osteoblasts (8, 11). Thus, in 1991, Arnold I. Caplan refers to the stromal cells as stem cells and named them MSCs (12). MSCs are now identified in a variety of postnatal tissues, such as adipose tissue, umbilical cord blood, umbilical cord, dental pulp, periodontal tissue, and liver (13). However, the rapidly increasing knowledge of MSCs and their clinical trials makes it challenging to compare the study outcomes.

In 2006, the International Society for Cellular Therapy addresses minimal criteria to define human MSCs (14); MSCs exhibit plastic adherent and express surface antigens positive to CD105, CD90, and CD73 and negative to CD45, CD34, CD14 or CD11b, CD79a or CD19, and human leukocyte antigen DR. MSCs are capable of differentiating into mesenchymal cells including adipocytes, chondrocytes, and osteoblasts. Currently, this criterion has been well-recognized and helpful for the characterization and application of MSCs in pre-clinical and clinical studies.

## 3 Mechanisms of actions of MSC-EV therapy in osteopenia

Clinical applications of systemic MSC transplantation have been explored in various diseases (15). The mechanisms of MSC therapy were explained by the *in vitro* and *in vivo* properties of multipotency or immune modulation of donor MSCs at the recipient disease sites. Especially the osteogenesis of MSCs promotes several investigations of MSC therapy in osteopenia model animals and demonstrates the bone regenerative outcomes (16).

However, a sufficient number of systemically transplanted MSCs fail to engraft in recipient target tissues (17, 18), a critical question of why single transplanted MSCs exert long-term effects without the direct potency of donor MSCs.

### 3.1 Properties of MSC-EVs

EVs are bilayered lipid membrane-bound small vesicles released from their parent cells, such as MSCs, and classified into two subtypes, including exosomes and microvesicles (19, 20). Exosomes are endosome-derived vesicles with a diameter of 30–150 nm. Microvesicles, also called ectosomes, are generated from the plasma membrane as budded vesicles with a diameter of 100 nm–1 μm. Apoptotic bodies are produced when cells are induced cell death. The size of them is 50 nm to several μm in diameter. According to the minimal information for the study of EVs (21), EVs express membranous antigens, such as CD9, CD63, and CD81.

EVs contain a variety of bioactive molecules, including nucleic acids, such as messenger RNAs, micro RNA (miRNA), and long noncoding RNA (lncRNA), lipids, and proteins, indicating that EVs perform as cargo for transferring their intercellular signals to recipient cells and control the functions of recipient cells (5, 6).

Recent several studies revealed the mechanism of actions of systemic MSC therapy for osteopenia; the transplanted donor MSCs can release paracrine factors within MSC-EVs and functionally activate recipient cells by transferring therapeutical signal(s) *via* donor MSC-EVs, leading to ameliorate the reduced bone mineral density (22).

Apoptotic bodies (ApBs) of donor MSCs play crucial roles in MSC-based therapy through the intercellular communications between transplanted donor MSCs and recipient cells (23). Donor MSC-mediated T lymphocyte apoptosis *via* FasL/Fas pathway is proposed as a mechanism of MSC therapy (24). Systemic infusion of exogenous ApBs of BMMSCs ameliorates bone mineral reduction in several osteopenia models, including MRL/*lpr*, Caspase 3^-/-^, and OVX mice (25). The infused ApBs activate the Wnt/β-catenin pathway in the recipient BMMCs by transferring ubiquitin ligase RNF146 and miR-328-3p within ApBs and regulate the differentiation of recipient osteoblasts and osteoclasts. Thus, the BMMSC ApB-mediated action is focused on the potential control of recipient bone metabolism to treat osteoporosis. However, there is no information on whether MSC-derived ApBs exhibit similar therapeutic benefits to osteopenia treatment of MSC-EVs. Further studies may be necessary to evaluate the effects and mechanism of MSC-derived ApBs in osteopenia treatment.

### 3.2 Target cells and therapeutic molecules in MSC-EV therapy for osteopenia

Numerous studies of MSC-EV therapy have been performed in osteopenia model animals (22, 26) (**Table 1**).

**Table 1:** Summary of parent cell origins, animal models, route and times of injection, actions, and target cells of MSC-derived extracellular vesicle therapy for osteopenia.

Parent cells and donor	Animal Model	Injection route and times	Action	Target cells	Ref.
BMMSCs, Human	BALB/c mice, OVX	IBM injection, two times	LncRNAs MALAT1 promotes osteoblast activity through SATB2.	Osteoblasts	(27)
BMSCs, Human	SD rats, OVX	IBM injection, twice a week, 3 weeks	MiR-935 promotes proliferation and differentiation in osteoblasts through STAT1 inhibition	Osteoblasts	(28)
Osteogenic UC-MSCs, Human	C57BL/6 mice, OVX	IP injection, every 3 days, 6 weeks	miRNAs are involved in osteogenesis and osteoclastogenesis through MAPK signaling.	Osteoblasts	(29)
AD-MSCs, Rat	SD rats, Hyperglycemia, STZ injection	IV injection, every two day, 6 weeks	Suppressing NLRP3 inflammasome activation in osteoclasts	Osteoclasts	(30)
AD-MSCs, Human	CD-1 mice, OVX	IV injection, twice a week for 2 weeks	OPG and MiR-21-5p inhibits osteoclastogenesis *via* RANKL.	Osteoclasts	(31)
AD-MSCs, Human	BALB/c mice, PGIA	IS injection, once a week for 6 weeks	MiR-21 inhibited osteoclast activation in spine.	Osteoclasts	(32)
iPSC-MSCs, Human	C57BL/6 mice, OVX	IV injection, once a week for 6 weeks	SiShn3 promotes osteogenesis and suppresses osteoclast activation *via* OPG/RANKL axis by osteoblasts.	Osteoblasts	(33)
UC-MSCs, Human	C57BL/6 mice, OVX	IV injection, two times	CLEC11A enhances osteogenesis of BMMSCs and inhibits osteoclastogenesis of monocyte.	BMMSCs, Monocytes	(34)
BMMSCs, Human	SD rats, OVX	IV injection, once a week, 4 weeks	MiR-186 promote osteogenesis of BMMSCs through Hippo signaling.	BMMSCs	(35)
BMMSCs, Rat	SD rats, OVX	IV injection, once a week, 8 weeks	Glycoprotein non-melanoma clone B promote osteogenesis of BMMSCs.	BMMSCs	(36)
BMMSCs, Mouse	Cbst KO mice	IV injection, three times a week, 8 weeks	Lnc-H19 activates lnc-H19/Tie2-NO signaling in BMMSCs and ECs through Angpt1.	BMMSCs, ECs	(37)
BMMSCs, Mouse	MRL/*lpr* mice	IV injection, one time	Fas rescues osteogenesis of BMMSCs *via* miR-29b-Notch pathway.	BMMSCs	(38)
UC-MSCs, Human	SD rats, Hindlimb unloading	IM injection, one time	miR-1263 activates YAP in BMMSCs *via* Mob1.	BMMSCs	(39)
SHED, Human	C57BL/6 mice, OVX	IV injection, one time	RNA enhances osteogenesis and suppresses osteoclastogenesis through SEMA3A in BMMSCs.	BMMSCs	(40)
BMMSCs, Rabbits	Rabbits, OVX	IV injection, one time	Not applicable	Not applicable	(41)

ATF4, activating transcription factor 4; AD-MSCs, adipose-derived MSCs; ALP, alkaline phosphatase; Angpt1, angiopoietin 1; Bad, B-cell lymphoma 2 associated agonist of cell death; BMMSCs, bone marrow MSCs; CLEC11A, C-type lectin domain family 11, member A; EVs, extracellular vesicles; Hoxa2, homeobox A2; iPSC-MSCs, induced pluripotent stem cell-derived mesenchymal stem cells; IL-6, interleukin 6; IBM, intra-bone marrow; IM, intramuscular; IP, intraperitoneal; IS, intraspinal; IV, intravenous; lncRNAs, long non cording RNAs; MALAT1, metastasis associated lung adenocarcinoma transcript 1; MAPK, mitogen-activated protein kinase; miRNAs, micro RNAs; NO, nitric oxide; NLRP3, nucleotide-binding oligomerization domain-like receptor family pyrin domain-containing 3; OPG, osteoprotegerin; OVX, ovariectomized; PGIA, proteoglycan-induced ankylosing spondylitis; RANKL, receptor activator of nuclear factor kappa-B ligand; Runx2, runt-related transcription factor 2; shn3, schnurri-3; SEMA3A, semaphorin-3A; SD rats, Sprague–Dawley rats; STAT1, signal transducer and activator of transcription 1; siShn3, small interfering RNA for schnurri-3; SATB2, special AT-rich sequence-binding protein 2; SHED, stem cells from human exfoliated deciduous teeth; STZ, streptozotocin; Tie2, TEK receptor tyrosine kinase; UC-MSCs, umbilical cord MSCs; YAP, yes-associated protein.

#### 3.2.1 MSC-EVs target recipient osteoblasts and osteoclasts

MSC-EV-containing RNAs enhance the proliferation and bone formation of recipient osteoblasts; lncRNA of metastasis associated lung adenocarcinoma transcript 1 in MSC-EVs acts as a sponge of miR-34c to promote the alkaline phosphatase activity and calcified nodule formation of osteoblasts associated with the increased gene expression of special AT-rich sequence-binding protein 2, runt-related transcription factor 2, and activating transcription factor 4 and decreased gene expression of homeobox A2 (27). MiR-935-contained MSC-EVs promote the proliferation and differentiation of osteoblasts by inhibiting signal transducer and activator of transcription 1 expression (28). MiRNAs within MSC-EVs enhance the proliferation and osteogenesis of osteoblasts, while the osteogenic MSC-EVs do not affect the proliferation of osteoblasts (29).

MSC-EV contents, including RNAs and proteins, suppress the differentiation and activation of recipient osteoclasts; MSC-EVs suppress osteoclasts through nucleotide-binding oligomerization domain-like receptor family pyrin domain-containing 3 inflammasome activation (30). Osteoprotegerin (OPG) within MSC-EVs inhibits receptor activator of nuclear factor kappa-B ligand (RANKL)-induced osteoclast differentiation. Both miR-21-5p and let-7b-5p within MSC-EVs inhibit osteoclastogenesis (31). MSC-EV-containing miR-21 suppresses osteoclast activation, as indicated by decreased serum levels of tartrate-resistant acid phosphatase 5b and cathepsin K, and decreased expression of interleukin 6 in bone tissue (32).

Interestingly, siShn3 artificially loaded into EVs promotes osteoblast activity and suppresses OPG/RANKL axis-activated osteoclasts (33).

#### 3.2.2 MSC-EVs target recipient BMMSCs

Multiple injections of MSC-EVs acquire the therapeutic effects on osteopenia; MSC-EV-containing C-type lectin domain family 11, member A enhances the commitment of recipient BMMSCs from adipocytes to osteoblasts and inhibits osteoclastogenesis of recipient monocytes (34). MiR-186 within MSC-EVs promotes osteogenesis of recipient BMMSCs through Hippo signaling pathway (35). Glycoprotein non-melanoma clone B-enriched MSC-EVs promote osteogenesis of recipient BMMSCs (36). Lnc-H19 within MSC-EVs activates lnc-H19/TEK receptor tyrosine kinase-nitric oxide signaling in recipient BMMSCs *via* angiopoietin 1, leading to inducing bone formation (37).

A single administration of MSC-EVs exerts bone recovery; MSC-EVs provide Fas to rescue *Fas*-deficient osteogenesis of recipient BMMSCs *via* regulating miR-29b-Notch pathway (38). Moreover, MSC-EVs can inhibit the apoptosis of recipient BMMSCs by transferring miR-1263 to inhibit the Hippo-mediated signaling pathway (39).

Taken together, the action of singly administrated MSC-EVs may cause the epigenetic memorization of stemness, including self-renew and cellular functions, in recipient tissue-specific MSCs and exert the long-term recovery and maintenance of bone metabolism. Meanwhile, multiple injections of MSC-EVs for osteopenia treatment may exert undesirable inconvenience to the patients of osteopenia in the clinical application of MSC-EV therapy, suggesting that single MSC-EV administration, targeting recipient tissue-specific MSCs, may be clinically safe and beneficial application for osteopenia.

## 4 A novel mechanism of MSC-EV therapy through microenvironmental modulating function of recipient BMMSCs

In the following sections, we will focus on the microenvironmental modulating function of recipient BMMSCs, which include bone metabolism interplayed with osteoblasts and osteoclasts, as an action of MSC-EV therapy.

### 4.1 Microenvironmental modulating function of MSCs

MSCs have been known to interact with neighboring cells directly (cell-to-cell contact) and indirectly (paracrine factors, EVs, and apoptosis) and modulate the microenvironment for immune cells and other tissue-specific cells, suggesting that tissue-specific MSCs potentially act as tissue-specific microenvironmental modulators in recipients after transplantation (23): in bone and bone marrow, resident BMMSCs can communicate with microenvironmental cells directly and indirectly and participate in stem cell niche formation, bone marrow hematopoiesis (42), and microenvironmental reorganization regulated by osteoblasts and osteoclasts (43, 44).

**Figure 1:**
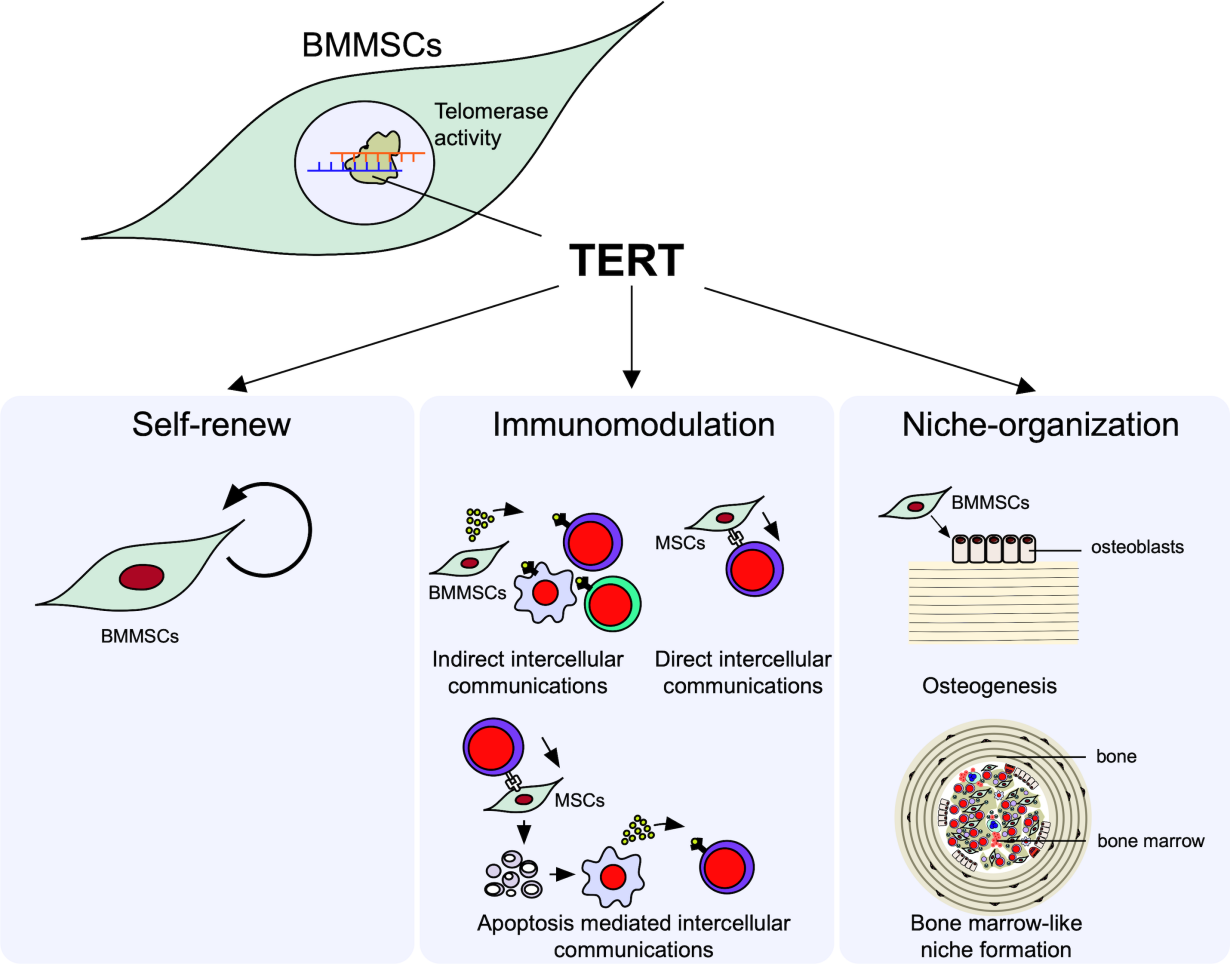
Microenvironmental modulation of bone marrow mesenchymal stem cells (BMMSCs). Telomerase reverse transcriptase (TERT) mediated telomerase activity in BMMSCs regulates their self-renew and microenvironmental modulation, including immunomodulatory and niche organizing functions. BMMSCs exhibit the self-renewal capacity to maintain their stemness. BMMSCs exhibit immunomodulatory function; BMMSCs can suppress the differentiation of interleukin 17-producing helper T (Th17) cells and induce the differentiation of regulatory T cells (Tregs) in directly (cell-cell-contact) and indirectly (paracrine factors and extracellular vesicles [EVs]) manners. BMMSCs can differentiate into osteoblasts and form *de novo* lamellar bone matrix. BMMSCs can conduct recipient derived bone marrow cell-like hematopoietic cells and organize hematopoietic niche surrounded by bone matrix.

Here, we focus on two properties of immune modulation and niche-organization as the microenvironmental modulating function of MSCs (**Figure 1**).

#### 4.1.1 Immunomodulatory properties of MSCs

MSCs exhibit an immunomodulatory property to a variety of immune cells, such as T lymphocytes, B lymphocytes, and macrophages (45, 46). The immunosuppressive functions of MSCs are explained by the following three mechanisms: 1) indirect intercellular communications with innate and adaptive immune cells by paracrine factors, including growth factors, cytokines, and container within EVs, such as miRNA; 2) direct intercellular communications with T lymphocytes through cell surface molecules, such as FasL and program death ligand 1; 3) efferocytosis of T lymphocyte-induced ApBs of MSCs, followed by secreting immunosuppressive factors from phagocytes (23, 47).

#### 4.1.2 Niche-organizing properties of MSCs

When BMMSCs are subcutaneously implanted with hydroxyapatite/beta-tricalcium phosphate (HA/TCP) as a carrier into immunocompromised mice, donor BMMSC-derived osteoblasts deposit *de novo* lamellar-bone-like matrix on the carriers (48). Interestingly, in a space surrounded by the *de novo* bone-like matrix, mononuclear cells exhibit hematopoietic properties of hematopoietic colony formation and expression of hematopoietic cell markers, including Sca-1 and c-Kit stem cell markers and CD45 lymphoid progenitor cell marker (49, 50). The mononuclear cells can also systemically circulate in the recipient body, indicating that the *de novo* microenvironment is implicated in a hematopoietic niche, likely in the bone marrow. These findings indicate that BMMSCs potentially serve as hematopoietic niche-organizing cells, as well as bone-forming cells, and contribute to the regulation of lymphocyte production and differentiation.

#### 4.1.3 Telomerase reverse transcriptase and telomerase activity regulate the microenvironmental modulating function of BMMSCs

MSCs are defined by two important properties of self-renewal and multipotency into various types of functional cells, as likely to embryonic stem cells (ESCs) (51, 52). MSCs undergo asymmetric mitotic divisions and become two daughter cells. One daughter cell maintains the properties of MSCs, while the others undergo further symmetric division to produce more progenitor cells of tissue-specific cells. Thus, MSCs can provide an internal repair system in the human body by recruiting tissue-specific cells into damaged tissues and organs. MSCs must acquire the sustained self-renewal and proliferative capacity to maintain homeostasis in the human body (13).

Telomerase reverse transcriptase (TERT) is a catalytic subunit of telomerase and is essential to acquire telomerase activity in cells (53). Suppression of TERT inhibits the pluripotency and differentiation of human ESCs; meanwhile, ectopic TERT expression enhances the colony-forming ability, proliferation, and differentiation of ESCs (54) and BMMSCs (55).

Telomerase plays critical roles in stemness of stem cells (54). Telomerase reactivation in adult stem cells can help to repaired of tissue degeneration (56). Telomere attrition is considered one of the hallmarks of epigenetic changes in aging stem cells (57). Furthermore, in nuclear reprogramming process of induced pluripotent stem cells, telomere rejuvenation is occurred by regulating epigenetic modification on chromatin and DNA (58).

Telomerase activity is detectable in HSCs and MSCs, but the levels are lower than those in ESCs. The moderate levels of telomerase activity in HSCs are essential to support the rapid turnover of differentiated blood cells (59). Furthermore, telomerase knockout mice express several disorders (60, 61). Telomerase dysfunction impairs the function and engraftment of HSCs (62). Moreover, TERT-deficient mice exhibit bone loss by suppressing osteoblasts and accelerating osteoclasts (63).

Thus, TERT is a critical rate-limiting component to control telomerase activity for maintaining and regulating the sustained self-renewal, proliferation, and functions of MSCs. In other words, TERT is considered a significant key enzyme to control the microenvironmental modulating function of MSCs.

### 4.2 Impaired microenvironmental modulating function of recipient BMMSCs in osteopenia model mice

**Figure 2:**
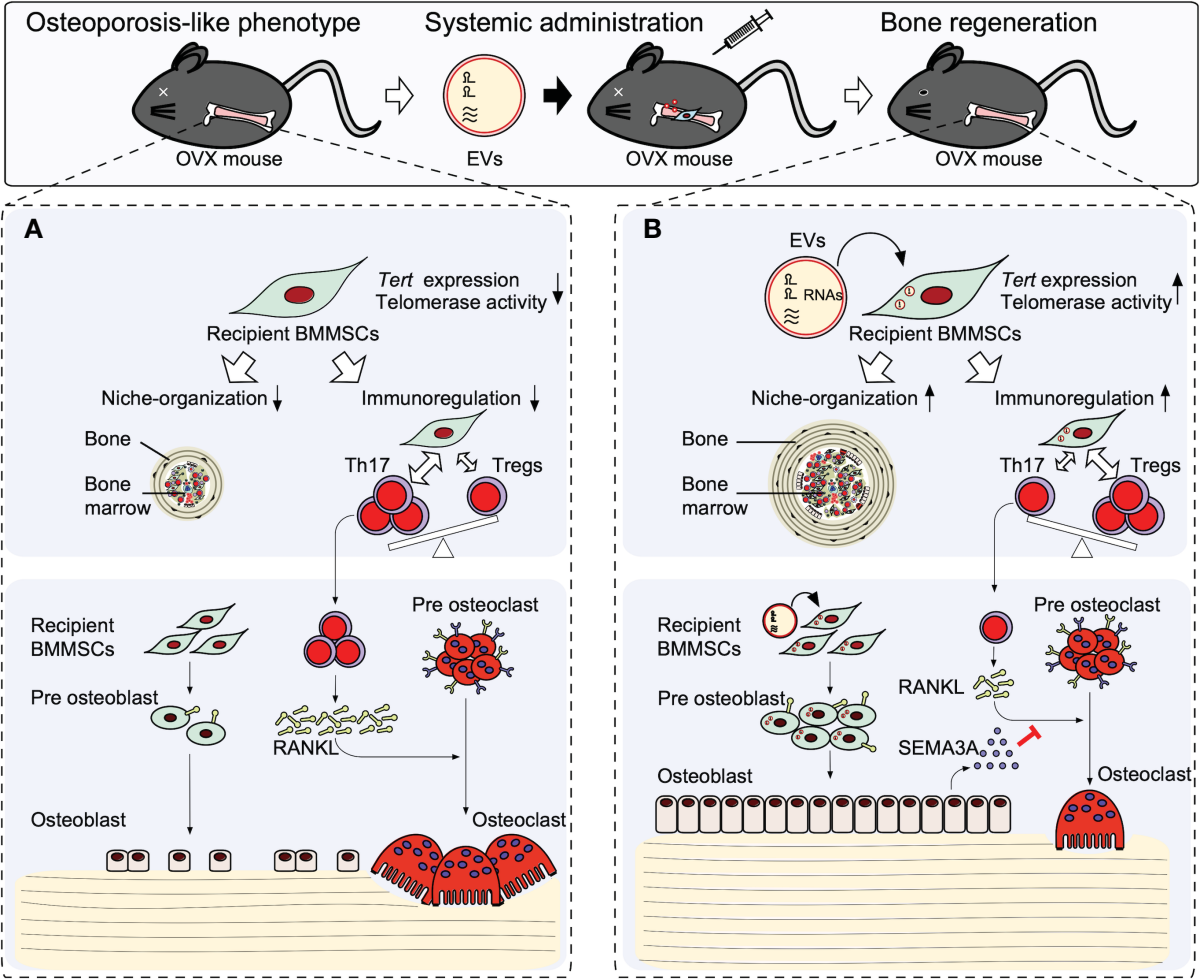
Therapeutic effects of stem cells from human exfoliated deciduous teeth releasing extracellular vesicles (SHED-EVs) on osteopenia model mice. Systemic administration of SHED-EV ameliorates osteopenia phenotype. **(A)** In osteopenia bone marrow, recipient BMMSCs exhibit an impaired microenvironmental modulating function including bone marrow-like niche formation and immunosuppressive regulation associated with the reduction of telomerase reverse transcriptase (*Tert*) gene expression and telomerase activity. The impaired recipient BMMSCs causes the direct suppression of osteogenesis and induced osteoclast differentiation *via* activated Th17 cells, resulting in causing bone loss. **(B)** Systemic administrated SHED-EVs transfer RNA contents into recipient BMMSCs. Mechanistically, RNA contents within SHED-EVs epigenetically enhance the telomerase reverse transcriptase gene (*Tert*) expression and telomerase activity in recipient BMMSCs. Finally, the activated telomerase activity rejuvenates the environmental modulating function of recipient BMMSCs, subsequently recover the impaired osteogenesis and semaphoring 3A (SEMA3A) releasing of recipient BMMSCs. The upregulated SEMA3A inhibit osteoclast differentiation *via* receptor activator of nuclear factor-κB ligand (RANKL).

Recipient BMMSCs isolated from non-treated osteopenia model mice (recipient OPe-BMMSCs) display deficient MSC stemness of self-renewal and osteogenic capacity. In addition, the recipient OPe-BMMSCs exhibit a suppressed immunomodulatory function to interleukin 17 helper T lymphocytes (Th17) and suppressed regulatory T lymphocytes (Tregs). Moreover, the recipient OPe-BMMSCs display damaged hematopoietic functions, including the *de novo* formation of bone marrow-like hematopoietic niche and *in vitro* induction of hematopoietic colony formation (40). Thus, the recipient OPe-BMMSCs may be epigenetically damaged in the stemness, microenvironmental modulatory function, and osteogenic ability (**Figure 2**). Therefore, the dysfunctions of recipient OPe-BMMSCs are considered one of the critical pathogenesis in osteopenia.

### 4.3 Systemic administration of stem cells from human exfoliated deciduous teeth-derived EVs target the recipient BMMSCs to rejuvenate the microenvironmental modulating function *via* their telomerase reverse transcriptase/telomerase activity in osteopenia treatment

#### 4.3.1 Application of stem cells from human exfoliated deciduous teeth and stem cells from human exfoliated deciduous teeth-derived EVs

Twenty years ago, Songtao Shi accidentally discovered stem cells from human exfoliated deciduous teeth (SHED) in the remnant dental pulp tissue of his daughter’s exfoliated deciduous teeth and characterized them as neural crest-derived MSCs (64). In addition to the well-defined mesenchymal immunophenotype and multipotency, SHED can exhibit multiple ectodermal and endodermal lineage cells, such as oligodendrocytes (65), dopaminergic neurons (66), endothelial cells (67, 68), insulin-producing cells (69), hepatocytes (70), and cholangiocytes (71). Furthermore, SHED display the self-renewal capacity (64, 72) associated with the low level of telomerase activity (72). Disease-specific SHED are also discovered and rejuvenated by pharmacological approaches (73–75).

Single systemic transplantation of SHED provide therapeutic effects on autoimmune disease (72, 76), chronic liver fibrosis (77), and entero-neuropathy (78). Recently, protocols for manufacturing quality-controlled clinical-grade SHED have been established (79–81). SHED demonstrate less tumorigenicity, low immunogenicity, and relative cryopreserved stability (76). Indeed, current studies report the clinical benefits of SHED therapy in human diseases, including traumatic dental pulp tissue and type 2 diabetes (82, 83). Thus, SHED therapy are gradually achieving a promising option for treating human diseases (84).

Increasing studies report SHED-EVs carry *in vitro* and *in vivo* biological actions of SHED (47, 85). SHED-EV-containing various bioactive molecules, such as nucleic acids and protein, are transported to target cells selectively by an unknown mechanism, resulting in implicating cell-cell communication, signal transduction, immune modulation, and epigenetic reprogramming of recipient cells. Recently, the benefits of SHED-EV administration are evaluated in bone regeneration (86), neuroprotective effects (87, 88), and anti-inflammatory function (89, 90) in disease model mice.

#### 4.3.2 SHED-EVs are mechanistically involved in systemic transplantation of SHED for osteopenia treatment

The systemic transplantations of SHED rescued the osteopenia phenotype as indicated by the significant increase in bone mineral density and trabecular bone structure (2, 72, 76). The single SHED transplantation improved the reduced osteogenic capacity of recipient BMMSCs and suppressed the differentiation and bone resorptive activity of recipient osteoclasts. The systemic transplantations of SHED also recovered the abnormal immune reactions of enhanced Th17 cells and suppressed Tregs in the osteopenia model mice. Interestingly, the single systemic transplantations rescued the microenvironmental modulating function of forming *de novo* bone and bone marrow by recipient BMMSCs, indicating that SHED therapy can exert therapeutic effects on osteopenia phenotype.

Meanwhile, the very low frequency of donor SHED are engrafted in the recipient bone and bone marrow tissues in osteopenia model animals (2, 72, 76). Further systemic infusion of SHED-derived conditioned medium exert the bone regeneration in OVX mice (91), suggesting that the indirect manner of transplanted donor SHED is involved in the improvement of the reduced bone mineral density (25, 31).

Systemic SHED-EV administration ameliorates the recovery of bone loss associated with osteoclast activation and hyperactivation of Th17 cells in the osteopenia model mice (40, 92), indicating that SHED-EV therapy is an alternative option for treating osteopenia and may target the recipient BMMSC function(s).

In the following literature, we discuss a unique mechanism of action of SHED-EV therapy for osteopenia treatment, which rejuvenate the bone marrow microenvironment through recipient BMMSCs (40, 47, 92).

When SHED-EVs are systemically administrated into osteopenia model mice, SHED-EVs are up-taken in the recipient OPe-BMMSCs and the microenvironmental modulating dysfunction of recipient OPe-BMMSCs are improved. The SHED-EV administration rescues the reduced *Tert* expression and reduced telomerase activity of recipient OPe-BMMSCs. Systemic administration of RNase-preconditioned SHED-EVs mostly attenuated the phenotypical and cellular effects of SHED-EV administration.

Small RNA, such as microRNAs, is well-known to epigenetically regulate gene expression (93, 94). MIR346 can bind to a region in the 3’UTR of TERT mRNA, leading to upregulating TERT expression (95, 96). In fact, SHED-EVs contain MIR346, and when SHED-EVs are incubated with human BMMSCs, MIR346 is increased in human BMMSCs. Therefore, SHED-EV-derived MIR346 may act as a candidate to regulate *Tert* expression and telomerase activity epigenetically, leading to participation in SHED-EV therapy for osteopenia (**Figure 2**).

Semaphorin 3A (SEMA3A) plays an osteoprotective factor produced by osteoblasts (97, 98) and inhibits osteoclast differentiation *via* receptor activator of nuclear factor-kB ligand by binding to neuropilin-1. The recipient osteogenic OPe-BMMSCs show the suppressed expression of SEMA3A and exhibit the decreased capacity for mineralized tissue deposition. Systemic SHED-EV administration increases the mineralized tissue deposition associated with the increased expression of SEMA3A in the recipient OPe-BMMSCs. The hyperactivated osteoclasts in osteopenia model mice are suppressed by the SHED-EV administration. Meanwhile, RNase-preconditioned SHED-EV administration attenuates the SHED-EV efficacy to the expression of SEMA3A in the recipient OPe-BMMSCs and osteoclast suppression.

Taken together, systemic administration of SHED-EVs exerts a therapeutic effect in osteopenia by improving the microenvironmental modulating function of recipient BMMSCs through enhancement of *Tert* expression and telomerase activity by SHED-EV-transferred miRNA. Thus, the rejuvenated recipient BMMSCs mainly contribute to bone reconstruction by regulating the function of osteoblasts and osteoclasts through SEMA3A (**Figure 2**).

## 5 Summary and future challenge

On regenerative treatment for osteopenia, MSC therapy is a promising option to recover bone mineral reduction by regulating the balance between osteoclast and osteoblast activity and immunomodulation. MSC-EV-mediated intercellular signal communications play an important role in MSC therapy. On the other hand, MSC-EV therapy is considered the alternative to MSC therapy for treating osteopenia. The common mechanism in MSC-based and MSC-free therapies is that MSC-EVs transfer a signal(s) to rejuvenate the impaired functions of recipient BMMSCs; this process is accompanied by epigenetic changes of telomerase activity in the recipient BMMSCs. Following that, the impaired recipient BMMSCs can acquire normalized self-renewal, stemness, and function to reconstruct the destructed bone matrix and maintain bone homeostasis. Once the self-renewal properties are epigenetically rescued in recipient BMMSCs, especially through TERT-telomerase pathway, the therapeutic effects in the recipient tissue may last in the long term. Thus, the epigenetic regulation in recipient BMMSCs is a promising target for systemic degenerative diseases, such as osteopenia. Further investigations, which are focused on epigenetic regulations in recipient tissue-specific MSCs, will open the novel door for establishing a safe and highly effective therapy not only for osteopenia but also for systemic degenerative disorders.

Generally, EVs cannot proliferate themselves (4, 21) and can circulate systemically without tissue trapping (18, 99, 100). Meanwhile, tumorigenesis and pulmonary embolism are major risks in general MSC therapy. MSC-EVs exhibit stable components under cryopreserved and freshly thawed conditions without losing their functional properties, while cryopreserved MSCs significantly lose their immunomodulatory properties and must be cultured to recover their full properties (101, 102). Thus, due to the safety and practical and functional off-the-shelf option, MSC-EV therapy may have a promising potential compared to MSC therapy.

Despite the advantages of MSC-EV therapy, there are several challenges to achieve MSC-EV therapy as a standard option for osteopenia. Clinically, the large-scale manufacturing of MSC-EVs is a considerable matter for delivering to many patients. As the quantity of MSC-EVs affects the quality, the consistent reproducibility of therapeutic effects on osteopenia relies on the quality of MSC-EVs. Therefore, to make MSC-EV therapy successful, the quality control of MSC-EVs must be primally overcome. However, the quality control of MSC-EVs is much more challenging due to the heterogeneity of MSC-EV, source difference, and donor difference of the parent MSCs (22). Although further evaluation of the mechanisms of action by MSC-EVs for osteopenia treatment will be required, once the definitive molecular mechanism is identified, the bioactive factor(s) within MSC-EVs will enable us to use it as the key molecule for quality control. Further engineering may produce artificial nanoparticles that contain enough key molecules for osteoporosis treatment in the future.

## Author contributions

Conceptualization: TY and SS. Writing—original draft preparation: TY and SS. Writing—review and editing: TY and SS. Funding acquisition: TY and SS. All authors read and agreed to the published version of the manuscript. All authors contributed to the article and approved the submitted version.
